# Endovascular stenting of spontaneous isolated dissection of the superior mesenteric artery: A case report and literature review

**DOI:** 10.1097/MD.0000000000008598

**Published:** 2017-11-17

**Authors:** Dong-Na Gao, Qing-Hui Qi, Ping Gong

**Affiliations:** a Emergency Department; b Department of Abdominal Emergency, the First Affiliated Hospital of Dalian Medical University, Dalian, China.

**Keywords:** computed tomography angiography, endovascular stenting, spontaneous isolated dissection of superior mesenteric artery

## Abstract

**Rationale:**

Spontaneous isolated dissection of the superior mesenteric artery (SID-SMA) is a rare arterial disease that is difficult to differentiate from other diseases because of lack of specific clinical manifestation and for which there is no available optimal management strategy.

**Patient concerns:**

A 58-year-old male patient visited our emergency room with sudden onset of moderate-severe epigastric abdominal pain of uncertain cause.

**Diagnoses:**

Computed tomography scanning showed a characteristic “double lumen sign” of the superior mesenteric artery, and further computed tomography angiography findings revealed a dissected segment of the superior mesenteric artery.

**Interventions:**

Conservative management was administered for 5 days, but the abdominal pain remained. Subsequently, an endovascular stent was placed in the affected superior mesenteric artery. Postoperative antiplatelet therapy was administered for 6 months.

**Outcomes:**

The abdominal pain was relieved. Six months later, a follow-up of computed tomography angiography showed that the stent placed had no interval narrowing.

**Lessons:**

Based on our review and the illustration of this case, endovascular stenting may be a preferred rescue treatment in SID-SMA patients for whom initial conservative treatment fails.

## 1 Introduction

Spontaneous isolated dissection of the superior mesenteric artery (SID-SMA) is defined as a dissection of the superior mesenteric artery (SMA) without concomitant alteration of the aorta, celiac artery, inferior mesenteric artery, or renal artery. This is a rare but potentially fatal arterial disease, and its incidence has been underestimated. Recently, SID-SMA has been detected more frequently because of the increasing use of computed tomography angiography (CTA) for the evaluation of abdominal symptoms.^[1,2]^ Three possible therapeutic strategies, namely conservative treatment,^[3–5]^ surgical revascularization,^[6]^ or endovascular therapy,^[7–9]^ have been described. However, till date, no consensus or guideline on the best treatment for SID-MSA has been established. Here, we report a case of SID-SMA successfully treated with endovascular stenting along with a literature review.

## 2 Case report

**Figure 1:**
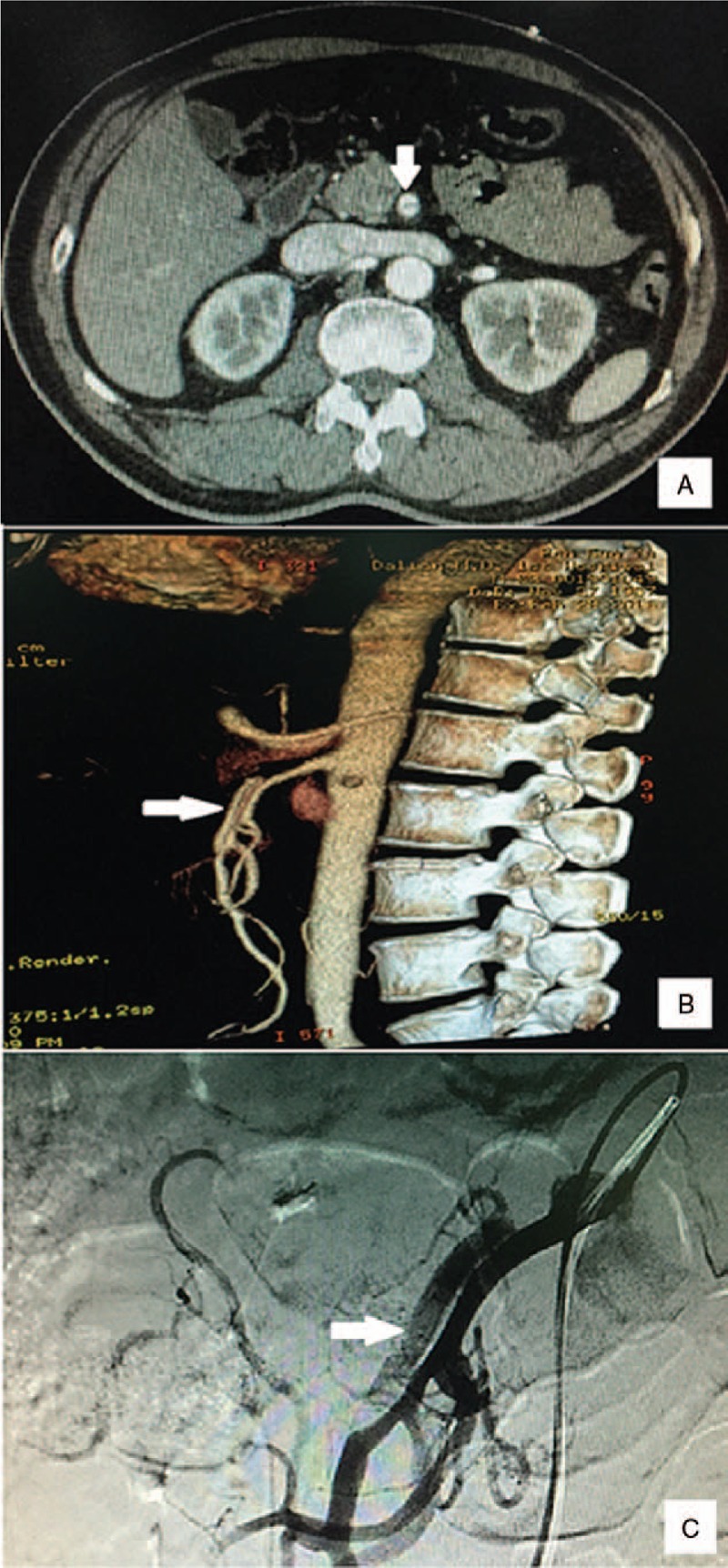
Diagnosis of spontaneous isolated dissection of the superior mesenteric artery. The characteristic finding of a “double lumen sign” of the superior mesenteric artery was found on axial views of computed tomography images (A), and a dissected segment of superior mesenteric artery was found on computed tomography angiography (B) and selective mesenteric angiography (C).

**Figure 2:**
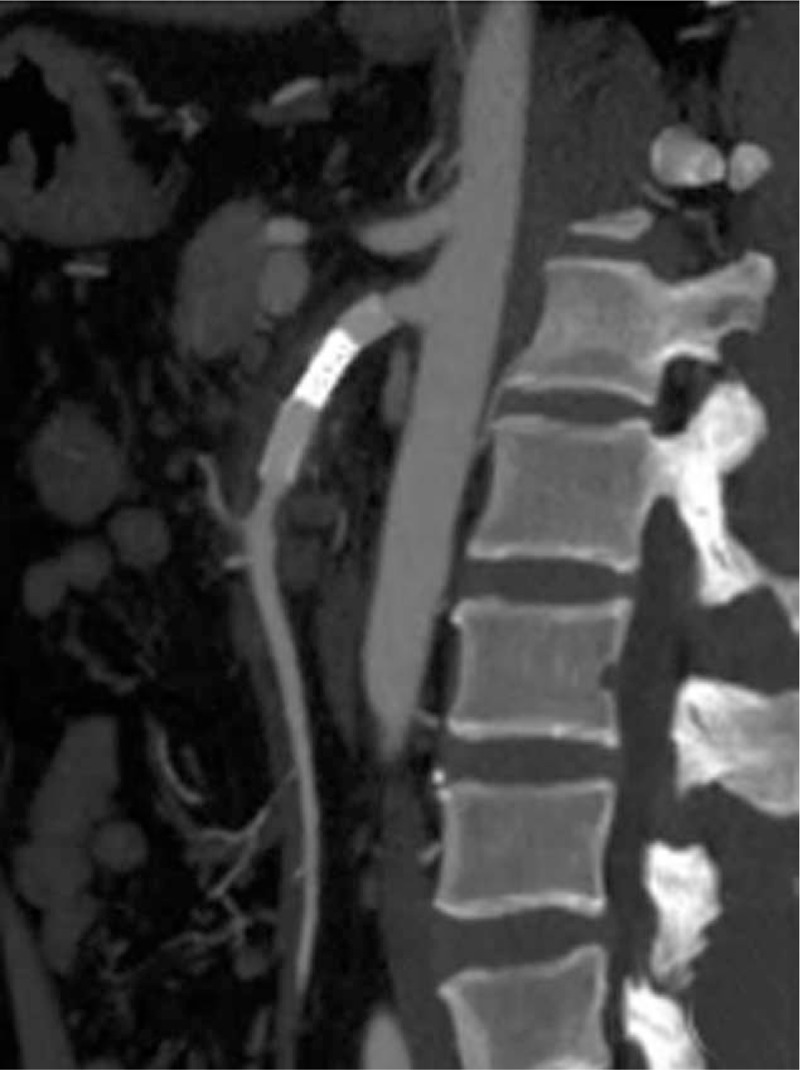
Computed tomography angiogram showing patency of the stent (arrow) 6 months postoperatively.

The study has been approved by the ethics committee of the First Affiliated Hospital of Dalian Medical University. Informed consent was obtained from the patient. A 58-year-old man had a sudden onset of persistent moderate-to-severe abdominal pain around the navel that slightly radiated to his back accompanied by headache, dizziness, and diaphoresis when taking a train. After 4 hours, he visited the emergency room of the First Affiliated Hospital of Dalian Medical University alone. His abdominal pain score was about 8/10 on the verbal quantitative scale. The patient had a past history of hypertension for over 10 years and smoking for 30 years. On arrival, he had an elevated blood pressure (230/120 mmHg) and increased pulse (110 beats/min). His respiration rate was 25 times per minutes, body temperature was 36.3°C, and oxygen saturation on breathing ambient air was 95% to 100%. He lay in a right lateral position but not a supine position because of his severe abdominal pain. No positive signs were found in lungs and heart. His abdomen was flat and soft without tenderness, tension, or rebound pain. The bowel sounds were decreased by 2 times/minute. Results of laboratory tests, including blood tests, coagulogram, glucose, electrolyte, cardiac troponin I, liver and kidney functions, were within normal ranges. The patient received intravenous injection of nicardipine (5 μg/kg/min) to decrease the blood pressure for 1 day followed by oral administration of nicardipine 10 mg daily till discharge, and a muscular injection of anisodamine (10 mg) to relieve the abdominal pain based on a suspected diagnosis of gastric spasm. Considering that the pain was not relieved after the above treatments, a computed tomography (CT) scan of the whole abdomen was performed and showed the characteristic “double lumen sign” of the SMA on the axial view of CT images (Fig. 1A). Next, abdominal aorta CTA was performed to rule out vascular disease, which revealed a dissected segment of SMA (Fig. 1B). Thus, the patient was admitted to the emergency department with a primary diagnosis of SID-SMA. Subsequently, conservative management was administrated, including strict blood pressure control (oral administration of nicardipine 10 mg daily), bowel rest, and close observation with oral anti-platelet agents (aspirin 100 mg and clopidogrel 75 mg daily). However, the abdominal pain was not relieved after 5 days of treatment. Selective mesenteric angiography was performed, and digital subtraction angiography (DSA) revealed a dissection in the SMA starting approximately 3 cm from its origin extending to the aortic orifice, with reduced blood flow to the intestine via the narrow SMA because of the narrowing of the dissecting aneurysm (Fig. 1C), which was classified as type IIa according to Yun's classification (Fig. 2).^[10]^ A 6 × 19 mm stent (Express LDTM, Boston Scientific Corporation, Natick, MA) was placed in the affected SMA. This stent placement resulted in a complete obliteration of the false lumen and in excellent distal blood flow, and thus, the abdominal pain was totally relieved. The patient was discharged after 10 days with good clinical prognosis. The antiplatelet therapy (aspirin 100 mg and clopidogrel 75 mg daily) was maintained for 6 months postoperatively, and the antihypertensive drug (oral nicardipine 10 mg daily) was continued. Follow-up CTA showed that that the stent placed had no interval narrowing without the recurrence of abdominal pain at 6 months postoperatively (Fig. 2).

## 3 Discussion

SID-SMA was first reported by Bauersfeld in the year of 1947.^[11]^ A study of 6666 autopsies reported an incidence of SID-MSA of 0.06%.^[12]^ The majority of cases have been found in Asia, the United States, Europe, and South America.^[1]^ This disease is more prevalent in men in their 50 seconds just like the patient we report herein.^[5,10,11]^ However, its etiology and pathogenesis remain unclear. Hypertension and smoking have been indicated as possible etiologies, which is consistent with the current case.^[1]^ Moreover, arterial wall pathology has been postulated as the underlying cause of SID-SMA, including arteriosclerosis, fibroelastic disease, fibromuscular dysplasia, cystic medial necrosis, medial degeneration of the arterial wall, adventitial inflammation, disruption of the internal elastic lamina, penetrating arterial ulcer, pseudoaneurysm, aneurysm, and trauma.^[1]^ Genetic heterogeneity of chromosome locus 5q13–14 has also been reported in 2 male familial cases of SID-SMA.^[13]^ An accepted hypothesis to explain the pathogenesis of SID-SMA is “shear stress injury” of the SMA.^[1,14]^ For this reason, the entry of the dissection is mostly located at the greater curve of SMA curvature (namely 1.5–3.0 cm from its origin) because the highest forces are borne in this area as a result of the transition of its fixed portion under the pancreas to its mobile segment at the mesenteric root.^[15,16]^

**Figure 3:**
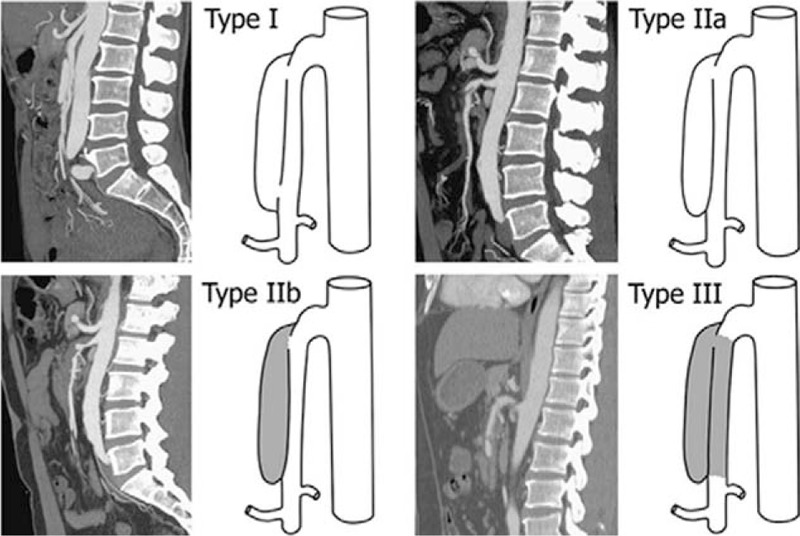
Angiographic classification of spontaneous isolated dissection of the superior mesenteric artery. Reproduced from Yun et al.^[10]^

The obvious clinical manifestation in most patients with SID-SMA is acute abdominal pain (mainly epigastric) combined with nausea, vomiting, or subacute intestinal obstruction. Moreover, some cases are asymptomatic, and these have been incidentally discovered and accounted for approximately one-fourth of SID-SMA patients.^[14,17]^ The abdominal pain is associated with mesenteric ischemia, stimulated visceral neuroplexuses resulting from the perivascular inflammation, or mesenteric hematoma.^[18–20]^ Pain severity is positively related to the dissection length.^[10]^ Therefore, it is difficult to make a definite diagnosis of SID-SMA because of lack of specific symptoms and signs, and thus, difficult to differentiate it from abdominal pain because of other diseases. Increasing clinical evidence shows that most patients with SID-SMA are identified by means of CT and magnetic resonance imaging (MRI) with the characteristic finding of a “double lumen sign” of the SMA on axial views, and CTA and selective mesenteric angiography with the characteristic finding of a dissected segment of the SMA.^[1]^ CTA is especially considered as a more suitable examination for the manifestation of dissection, thrombus (if any), and true and false lumens. According to the patency of the entry and re-entry sites, a simplified angiographic classification of SID-SMA was established by Yu et al^[10]^ which is based on Sakamoto's classification.^[20]^ A detailed description of the classification is as follows: patent true and false lumens with visible entry and re-entry sites are defined as type I; patent true and false lumens with visible entry but no visible re-entry (blind pouch of false lumen) are defined as type IIa; patent true lumen but thrombosed false lumen without visible re-entry is defined as type IIb, which usually causes true luminal narrowing; and confirmed SMA dissection with occluded true and false lumens is defined as type III (Fig. 3).^[10,20]^ However, neither Sakamoto et al^[20]^ nor Yun et al^[10]^ have observed a clear relationship between the clinical course and radiological appearance.

Till date, a consensus or guideline for the treatment of patients with SID-SMA has not been established. Most patients with SID-SMA may experience a self-limited course, but it is also potentially fatal because of major complications such as the rupture of the dissection or secondary intestinal ischemia and infarction resulting from stenosis of the SMA.^[2]^ Therefore, the aim of treatment for SID-SMA is to limit the extension of the dissection, prevent the rupture of the false lumen, and maintain the distal blood perfusion through the true lumen.^[21]^ In the clinical setting, the choice of the treatment strategies depends on the clinical presentation and the lesions observed on CTA.^[5,22,23]^ Currently available treatments for patients with SID-SMA include: conservative management consisting of strict blood pressure control, bowel rest, and close observation with or without the use of anticoagulation or anti-platelet agents; open surgery such as bypass or direct surgical reconstruction of the SMA lesion; or endovascular therapy with SMA stenting.^[23–25]^ Based on existing literature, if there are no clinical and imaging signs indicating ruptured SMA branches or bowel ischemia, most SID-SMA patients can be managed conservatively with a good outcome.^[20,26–28]^ However, presence of arterial rupture and intestinal infarction is an indication for open surgery.^[1,20,26–28]^ Endovascular therapy is suitable for symptomatic SID-SMA patient with type II lesions (with increased risk of further progression of the dissection) just as in the current case or secondary to failed conservative treatment with or without anticoagulation, especially in patients at high risk for surgery.^[22,23]^ In addition, for all hypertensive patients with SID-SMA blood pressure should be controlled within normal limits by intravenous or oral antihypertensive drugs. However, blood pressure of such patients should not be decreased too low to prevent from decreased blood supply and subsequent intestinal ischemia.

Endovascular stent placement for the treatment of SID-SMA was first described by Leung et al.^[29]^ Recently, endovascular treatment has been increasingly investigated in view of its characteristics of being minimally invasive and quick to implement along with its good safety and efficacy, just as in its application for most other vascular diseases.^[1,7–9,13,15,19,22,23,29–31]^ This treatment can provide immediate symptomatic improvement by obliterating the false lumen and increasing blood flow into the small intestine and prevent further progression of the SMA dissection in a short time.^[8]^ The choice of stent diameter is based on the proximal normal artery diameter. The types of bare stents are determined according to the operator's preference and the availability of the stent.^[7]^ Self-expanding stent placement via a right common femoral artery approach followed by antiplatelet therapy for 3 months postoperatively has been recommended because of its weaker radial force.^[9]^ The use of postoperative antithrombotic treatments for SID-SMA remains controversial. No apparent prognostic difference was observed between patients treated with or without postoperative antithrombotic medication.^[5,10,22]^ In general, patients who underwent endovascular bare stent placement are instructed to take aspirin and clopidogrel orally from 6 to 10 months postoperatively to prevent secondary thrombosis.^[8]^

However, there are still some limitations to endovascular treatment of SID-SMA. It is occasionally difficult to find the site at which tearing of the artery wall started during dissection of the SMA when endovascular stent placement is performed. Moreover, the long-term results, such as the risk of restenosis and re-occlusion of side branches of the stented segments in patients, have yet to be determined.^[1]^

In conclusion, SID-SMA is a rare condition that may be managed by conservative, surgical, or endovascular treatment based on clinical presentation. Endovascular stenting may be a preferred treatment in SID-SMA patients with moderate-to-severe symptoms or for whom initial conservative treatment has failed as a rescue strategy.

## Acknowledgments

The authors would like to thank Feng Wang, Rong-Sheng Liu, and Feng Li from the Interventional Department of the First Affiliated Hospital of Dalian Medical University for their help in treating the patient with endovascular stenting.
